# Critical methodological considerations in recruiting and engaging non-native English speaking workers with a head injury: a Canadian perspective

**DOI:** 10.1186/s13104-020-05028-y

**Published:** 2020-03-30

**Authors:** B. Nowrouzi-Kia, B. Sharma, J. Lewko, A. Colantonio

**Affiliations:** 1 Centre for Research in Occupational Safety and Health, Laurentian University, Sudbury, Canada; 2 Toronto Rehabilitation Institute-University Health Network, Toronto, Canada; 3 Centre for Research in Human Development, Laurentian University, Sudbury, Canada; 4 Rehabilitation Science Institute, University of Toronto, Toronto, Canada; 5 Department of Occupational Science and Occupational Therapy, Faculty of Medicine, University of Toronto, Toronto, Canada; 6 School of Nursing, Faculty of Health, York University, Toronto, Canada

**Keywords:** Methodological considerations, Traumatic brain injury, Qualitative, Interviews, Head injury, Occupational injury

## Abstract

**Objective:**

Non-native English speaking workers with a mild work-related traumatic brain and/or head injury are a vulnerable and underrepresented population in research studies. The researchers present their experiences with recruiting and performing qualitative interviews with non-native English speaking individuals with a work-related mild traumatic brain injury, and provide recommendations on how to better include this vulnerable population in future research studies. This paper presents considerations regarding ethics, recruitment challenges, interview preparation and debriefing, sex & gender and language and cultural issues must be made when working with this vulnerable population.

**Results:**

The researchers discuss critical issues and provide recommendations in recruiting and engaging with non-native English language workers including ethics, recruitment challenges, interview preparation and debriefing, sex & gender and language, and cultural considerations that must be made when working with this population. The study recommendations advise investigators to spend more time to learn about the non-native English participants in the mild wrTBI context, to be familiar with the vulnerabilities and specific circumstances that these workers experience. By increasing their awareness of the challenging facing this vulnerable population, the intention is to provide better care and treatment options through evidence-based research and practice.

## Introduction

In-depth qualitative interviews allow researchers to communicate with those who have knowledge of or experience with the problem of interest [1]. Through such interviews, researchers discover in detail the experiences, motivations, and opinions of others and learn to see the world from their perspectives [1]. Non-native English speaking workers are essential to include in occupational health research [2] because of their importance to the labour force in Canada and the economy [3], and because of their unique perspectives that may not be captured in occupational research involving native English speakers. For instance, from 2011 to 2016, some estimates suggest that immigrant workers account for all net labour force growth in the country [4]. Furthermore, the perspectives of immigrants are of particular importance to countries like Canada, where they comprise a significant segment of the labour force [5]. Moreover, immigrants working in physically demanding jobs may be exposed to particular risks of workplace injury [3] and have working conditions inferior to their native counterparts [6].

The purpose of this article is to outline, critically discuss, and identify challenges when working with non-native English speaking workers. The researchers share their experience with the intention to inform recruitment and engagement strategies for non-native English language workers in future research studies. The purpose of this study is to provide recommendations for doing research with non-native English speaking participants with examples from a study of persons with mild head trauma.

## Main text

### Methods

The study was conceptualized as a mixed-methods sequential explanatory investigation using a cross-sectional design with quantitative and qualitative elements. Data were collected through self-report questionnaires and through semi-structured interviews with workers who had experienced a head injury. The detailed methods and results are reported elsewhere [7, 8]. Our focus here is recruitment and engagement issues with non-native English workers during the qualitative phase of the study.

#### Participants in current study

In total, 32 workers, 35–64 years of age and who had sustained a head injury and/or had a formal diagnosis of a work-related TBI, were recruited from a neurology clinic in Ontario, Canada. In the larger sample, 102 participants were recruited, and 46.1% were female, 38.9% of workers had a prior history of a TBI and 37.1% had sustained an wrTBI [8]. The average time since injury was 15.9 ± 19.4 months [8].

Regarding the participation rate, the researchers had the names and contact information of 42 non-native English-speaking workers. However, the researchers recruited ten non-native English-speaking participants by phone with the assistance of an interpreter, providing us with a recruitment rate of 24% (the tenth participant was recruited in the clinic with a translator’s assistance). All of the participants spoke English as a second language.

All participant workers were referred to the clinic for an assessment for persistent symptoms associated with a work-related head and/or traumatic brain injury. Ten of the participants were non-native English speakers. All participants completed a self-report questionnaire (e.g. educational history, mechanism of injury, full-time or part-time employment status) and their medical records were reviewed to obtain additional information about their demographic and injury characteristics (e.g. age, clinical diagnoses, comorbidities).

In-depth semi-structured qualitative interviews were conducted by a research associate (BNK) who has training in qualitative interviewing methods and expertise in work-related head and/or traumatic brain injury. The interviews were either conducted in person (3) or by telephone (29), depending on the preference of the participant. Interviews with the ten non-native English speaking workers were conducted with the assistance of interpreters, and two were conducted in person and eight by phone. Each interview lasted approximately 60 min and was digitally recorded. For each of the ten interviews, an interpreter was available, regardless of whether the participant decided to use their services.

### Results

#### Ethical considerations

The study received research ethics approval. Accessing vulnerable populations for research purposes can be arduous, as participants must be protected from research that might be insensitive, intrusive, and potentially distressing [9]. Individuals referred for a work-related traumatic brain and/or head injury with persistent symptoms may be vulnerable including being new to the country, language barriers and unfamiliarity with their new occupation. Further, there are potential issues related to stigma that are culturally based (e.g., social stigma) [10].

Participants off work due to an occupational injury are considered a vulnerable population because of the risks related to disclosing sensitive information about their health and employment [11, 12]. For instance, one participant consented and agreed to participate in the interview but only after establishing trust with the researchers over a period of 8 weeks as a consequence of the injury. This worker was injured in a work environment where his trust in his co-workers was broken on several instances and which led directly to their injury, and initial distrust of research team members. Therefore, trust is a critical dimension of conducting a successful interview, and a vital element of qualitative inquiry [13] with vulnerable populations in particular. The researcher establishes trust by creating a supportive environment, using their knowledge of the topic (head injuries and the return to work process) and cumulative experience in the field of occupational health and safety.

As a recommendation (see Table 1), the researchers suggest that research hospitals in urban centers (serving multi-ethnic and lingual communities) provide the necessary resources (e.g., financial and human) to have certified site interpreters help in the recruitment on non-native English speakers to research studies by incorporating these costs into grant applications.

**Table 1:** Summary of challenges and recommendations for doing research with non-native English speaking participants

Challenge	Recommendation
Ethical considerations	Research hospitals in urban centers (serving multi-ethnic and lingual communities) provide the necessary resources (e.g., financial and human) to have certified site interpreters help in the recruitment on non-native English speakers to research studies by incorporating these costs into grant applications
Recruitment challenges	Provide participants the opportunity to discuss their story and experiences with a mild wrTBI. Moreover, they were given an occasion to contribute other issues that were not examined during the interview to empower them and provide them with an opportunity to raise pertinent issues for the research team’s consideration and to seek help
Interview preparation and debriefing	Establishing an advisory panel of TBI survivors and advocates for injured workers that can provide feedback and suggestions about the interviews, particularly in dealing with vulnerable workers
Sex & gender considerations	Sex and gendered considerations be incorporated when engaging with workers who are non-native English speakers and a vulnerable segment of the population
Language and cultural considerations	Early and frequent engagement with the interpreters before the interview with the non-native English-speaking worker. The interpreter should be provided with the study material in advance including information about the study and the researchers’ expectations regarding their role, contribution, and understanding of the interview process. Furthermore, specific language including a discipline’s jargon (e.g., use of medical terminology) should be explained in advance to provide the interpreter with an opportunity to prepare before the interview

#### Recruitment challenges

Successful participant recruitment is an important aspect of conducting qualitative research [14]. The participants were recruited from a consecutive sample of injured workers referred to an outpatient program at a large urban hospital in Ontario, Canada. This approach requires a considerable investment of resources including staffing, and data management and analysis. Regarding our participation rate, the researchers had the names and contact information of 42 non-native English-speaking workers. However, the researchers recruited ten non-native English-speaking participants by phone with the assistance of an interpreter, providing us with a recruitment rate of 24%.

The researchers identified several issues including obtaining consent, addressing concerns of confidentiality and anonymity and developing trusting relationships with participants, before conducting the interview and afterwards during the debriefing process. These safeguards were explained to the participants, and an opportunity for feedback and questions was provided.

Another recruitment challenge was recognizing the stigma associated with sustaining a mild work-related traumatic brain injury (wrTBI). Individuals who are stigmatized possess a devalued and denigrated identity in our society [15]. Participants were given the occasion to discuss their story and experiences with a mild wrTBI. Moreover, they were given an opportunity to contribute other issues that were not examined during the interview to empower them and provide them with an opportunity to raise pertinent issues for the research team’s consideration and to seek help.

#### Interview preparation and debriefing

Preparation and discussion for interviewing non-native English workers with mild work-related traumatic brain and/or head injury are vital and should include the research team. The research team made decisions regarding the interviewing process and worked collaboratively with the interviewer when working with informants whose first language was not English. The team met monthly and reviewed progress with the interviews, provided suggestions for improvement (e.g., the flow of the interview questions, transition from one topic to another) and discussed challenges to recruitment. The researchers recommend establishing an advisory panel of TBI survivors and advocates for injured workers that can provide feedback and suggestions about the interviews, particularly in dealing with vulnerable workers.

Reliability and credibility was ensured in several ways. The first involved the use of member checking after each interview. After the interview had been completed, the participant was provided with a summary and invited to make additions and/or changes for clarity and accuracy through an interpreter. This feedback from the participants was crucial to understanding their mild wrTBI. Furthermore, their consent was received to contact them if additional clarification or questions were warranted by the investigators. We recommend that this step is taken because it serves to demonstrate a genuine, trusting relationship with the participant and that the researchers are seeking to capture the intended meaning of their comments.

#### Sex & gender considerations

Women comprise a larger proportion of the workforce in Canada [16] and the United States [17]. However, there is a lack of evidence regarding sex and gender-specific issues among non-native English speaking workers with a mild wrTBI. This is especially important given that women are taking on workplace occupations with higher risk and with increasing frequency. A more equitable sex distribution is found with milder cases like concussions, which represent the fastest growing group of work-related traumatic brain and/or head injury claims. Women represent a growing number of lost-time injury claims in Ontario [18], and women are more likely to be filling roles that fall within the women’s unpaid labor (e.g., reproductive responsibilities, caregiving, domestic roles) [19–21]. Furthermore, this gendered nature of paid work has been related to return to work and receiving benefits after an occupational injury [20]. The initial interview guide contained a question related to sex and gender that stated “Do you think that your being a man/woman influenced your experience of brain injury?” However, the participants were perplexed, found it difficult to answer or stated that their sex and gender did not influence their lived experiences with a mild wrTBI. These concepts may also not translate well across cultures therefore, upon consultation with the research team, the question was revised to contain details about the roles, responsibilities, and relationships before and after their injury.

#### Language and cultural considerations

Evidence suggests that to conduct valid research, the investigator must be cognizant of personal and cultural perspectives or bias [22]. According to the cultural perspective, a threat may surface if the interpreter is not sufficiently trained, including the building of rapport with participants [2], or if the interpreter does not have a complete understanding of the particular research project, or has biased ideas [22]. The researchers had difficulties scheduling interpreters for in-person interviews, as they often did not know far enough in advance when a non-native English speaking participant was coming to the clinic where data collection occurred. Our recommendation is to have interpreters available on-site (sometimes with short notice) to assist with translation when interviewing a non-native English speaking participant.

During face-to-face interviews, the interpreter was seated beside the researcher on one side of a table. The researcher looked at the interpreter when directing a question, to ensure that the interpreter understood the question and was briefed before the interview. During phone interviews, the interpreter was informed before the interview regarding the nature of the interview, their role, the importance of respecting confidentiality and in having the privilege to listen to the individual’s narrative. With both settings, the interpreters were given the opportunity to debrief after the session. The interpreters in our study knew English, but only some may have had some experience serving as an interpreter in a research project. It was not possible to control for any bias in the interpreter [23]. Therefore, there were several threats to the validity of the project when the interviewer was working with the interpreter. A threat was when the researcher addressed a question in English to the interpreter since the researcher did not know how the interpreter perceived/and or/interpreted the question [23]. Particularly, questions related to how the mild wrTBI occurred required specific details from the informant regarding the nature, extent, and predictors of the injury.

## Limitations

Understanding the methodological challenges of interviewing non-native English speaking workers provides preliminary evidence to include these participants in clinical research. The inability to add this group of individuals can make present further challenges to access health care and make them vulnerable to exploitation [5]. The researchers have provided an overview of the issues and recommendations for conducting research with individuals with a mild work-related traumatic brain and/or head injury. Our study highlights challenges to conducting research with this important group of workers but also indicates that these are not impossible and can be overcome.

## Data Availability

The research data contains identifying/confidential patient data and cannot be shared. The data is stored securely and confidentially at the University Health Network (Toronot, Ontario, Canada).

## Abbreviation

wrTBI: Work-related traumatic brain injury
